# Morphological and Molecular Features of Porcine Mesenchymal Stem Cells Derived From Different Types of Synovial Membrane, and Genetic Background of Cell Donors

**DOI:** 10.3389/fcell.2020.601212

**Published:** 2020-12-09

**Authors:** Puntita Siengdee, Michael Oster, Henry Reyer, Torsten Viergutz, Klaus Wimmers, Siriluck Ponsuksili

**Affiliations:** ^1^Institute for Genome Biology, Leibniz Institute for Farm Animal Biology (FBN), Dummerstorf, Germany; ^2^Institute for Reproductive Biology, Leibniz Institute for Farm Animal Biology (FBN), Dummerstorf, Germany

**Keywords:** mesenchymal stem cells, SMSCs, synovial membrane, porcine synovium, German Landrace, Angeln Saddleback

## Abstract

Synovial mesenchymal stem cells (SMSCs) have become a great cell source for musculoskeletal stem cell research, especially related to cartilage and bone tissue regeneration, due to their superior cell proliferation properties and multidifferentiation potential into various cell lineages. This study revealed isolation methods, culture conditions, and morphological and molecular characterization of SMSCs derived fibrous synovium (FS) and adipose synovium (FP) of two pig breeds differing in growth performance [German Landrace (DL), and fat deposition (Angeln Saddleback (AS)]. Herein, FS possessed nucleated cell numbers nearly twice as high as those of FP at Passage 0. SMSCs derived from different types of synovial membrane and genetic background show similar cell morphologies and immunophenotypes, which were assessed by cell surface epitopes and multilineage differentiation potential, but differ significantly in their molecular characteristics. In addition, transcripts of SMSCs from AS were more enriched in IGF-1 signaling and VEGF ligand receptor, while SMSCs from DL were more enriched in growth hormone signaling and bone metabolism. The results indicate that genetics and tissues play significant roles for SMSC characteristics so that SMSCs can be traced back to the original cell donor and be used for fine turning in applications of medical research and therapies.

## Graphical Abstract

Flow chart of the experiment from cell isolation, culture conditions to morphological and molecular characterisation of synovial mesenchymal stem cells (SMSCs).

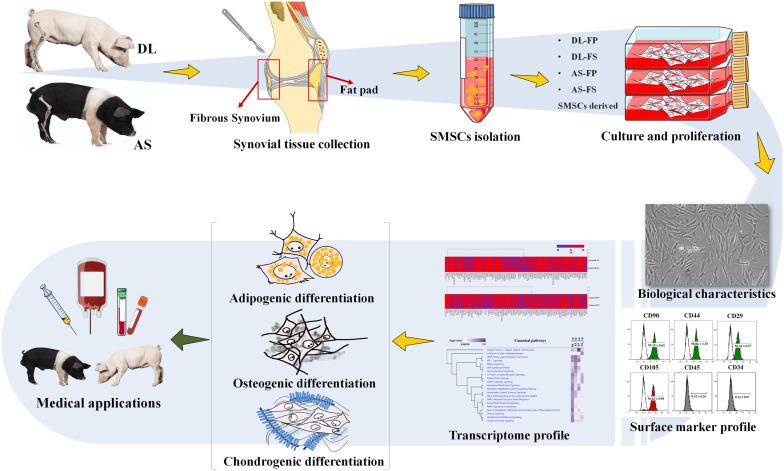

## Introduction

Multipotent and self-renewing mesenchymal stem cells (MSCs) have the potential to differentiate into various connective tissue cell lineages, such as osteocytes, adipocytes, chondrocytes, and even myocytes, under defined conditions (Mochizuki et al., 2006; Yamaguchi, 2014). MSCs provide a source for fascinating models of differentiation, cell therapy, and tissue engineering (Marion and Mao, 2006; Parekkadan and Milwid, 2010). MSCs can be generated from various adult tissues and organs, including bone marrow, muscle, adipose tissue, synovium, periosteum (Sakaguchi et al., 2005; da Silva Meirelles et al., 2006; Beane and Darling, 2012; Chong et al., 2012;

Baer, 2014; Rohban and Pieber, 2017). These MSCs are assumed to be similar irrespective of their original tissue sources and to have common surface epitopes (Yamachikaa and Iida, 2013). However, there is growing evidence that isolation rates, and functional properties of the cells, which influence their applicability, depend on the source from which they are harvested, as well as the preparation and differentiation techniques applied (Parekkadan and Milwid, 2010; Baer, 2014; Yamaguchi, 2014). Bone marrow and white adipose tissue are the main sources used to harvest MSCs because of their high isolation rate, high numbers of colony-forming units, and excellent cell properties (Yamaguchi, 2014; Xu et al., 2017; Mohamed-Ahmed et al., 2018). To date, synovium-derived cells have attracted significant interest as a potential source of MSCs and for clinical applications because their yield, expandability, proliferation potential, and differentiation potential are similar to, or even higher than, those of bone marrow, periosteum, adipose tissue, and skeletal muscle in humans (Wickham et al., 2003; Sakaguchi et al., 2005; Mochizuki et al., 2006; Alegre-Aguarón et al., 2012), rats (Yoshimura et al., 2007), and dogs (Sasaki et al., 2018). Studies on pigs confirmed the superior potential of synovium-derived MSCs in cartilage regeneration (Shimomura et al., 2010; Nakamura et al., 2012). However, preparation and isolation methods for synovium-derived MSCs (SMSCs) from domestic pigs have not been well described though representing valuable non-rodent models for medical research approximating human conditions.

Two types of synovial tissue can be classified on the basis of their anatomical relationship to the femur: one type overlies the non-cartilaginous areas of the medial and lateral femur (fibrous synovium); the other is present on the opposite side of the femur and covers the inner joint capsule (infrapatellar fat pad or adipose synovium), and is more easily accessible (Nishimura et al., 1999). In this study, we collected and expanded, using similar processes, both fibrous and adipose synovial tissue from two different pig breeds. By examining specific surface epitopes; different osteogenic, adipogenic, and chondrogenic differentiation potentials together with the differential gene expression profiles between breeds and two different types of synovial tissues this study aims to compare the properties of these cell populations to characterize the most suitable and accessible sources of pig SMSCs, which have not been well reported to date.

## Materials and Methods

### Ethics Statement

Animal care and tissue collection procedures were approved by the Animal Care Committee of the Leibniz Institute for Farm Animal Biology and carried out in accordance with the approved guidelines for safeguarding good scientific practice at the institutions in the Leibniz Association and the measures were taken to minimize pain and discomfort and accord with the guidelines laid down by the European Communities Council Directive of 24 November 1986 (86/609/EEC). For this study, the animals were used for meat production and underwent no experimental treatment, diagnostic sampling, or any other intervention before killing. Animal handling as well as the killing was in accordance with applicable laws, relevant guidelines, and provisions for ethical regulations.

### Collection and Preparation of Tissue Samples

**FIGURE 1:**
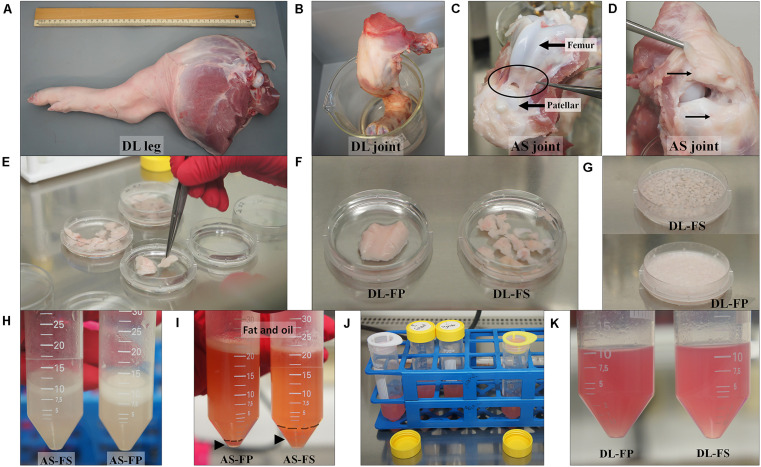
Tissue collection and isolation of porcine synovial mesenchymal stem cells (SMSCs). **(A,B)** Synovial tissues were harvested from German Landrace (DL) and Angeln Saddleback (AS) pigs’ stifle joints. **(C,D)** Fibrous synovium (FS) was harvested from the inner side of the lateral joint capsule; the suprapatellar bursa and adipose synovium (FP) were harvested from the inner side of the infrapatellar fat pad of the knee joint. **(E–G)** Synovial tissues were washed, chopped, and digested with collagenase. **(H–K)** After centrifugation, the cell suspension fraction was strained. The cell pellets were then resuspended in the growth medium and cultured.

The pig joints used in this study were from the hind legs (stifle joints) of three male 59 day old piglets of each of the German Landrace (DL, *n* = 3) and Angeln Saddleback (AS, *n* = 3) breeds. Following death, the pigs’ legs were carefully removed from the body at the acetabulum of the hip joint and immediately brought to a clean laboratory to remove the dirty skin and attached muscles taking care not to damage or open the joint capsule in this step avoiding contamination of synovium-derived cells. The stifle joints were soaked in 99.98% ethanol and brought to the cell culture laboratory (Figures 1A,B).

### Harvest of Synovial Tissue and Isolation of Synovium-Derived Mesenchymal Stem Cells

Synovial tissue collection and synovial mesenchymal stem cell isolation procedures previously described for dogs (Sasaki et al., 2018) and humans (Mochizuki et al., 2006; Katagiri et al., 2017) were modified to create the procedure used on pigs in the present study. Briefly, joint capsules were aseptically opened under a laminar flow hood. The porcine synovial membranes were removed from the knee joints. Two sources of synovial tissue were collected. Fibrous synovium (FS) was harvested from the inner side of the lateral joint capsule—especially at the suprapatellar bursa, which overlies the non-cartilaginous surfaces of the lateral condyles of the femur (Figure 1C). The adipose synovium (FP) was harvested from the inner side of the infrapatellar fat pad of the knee joint (Figure 1D). Synovial tissues were rinsed three times with PBS (Merck KGaA, Darmstadt, Germany) plus 10% antibiotic/antimycotic solution (Merck KGaA, Darmstadt, Germany), with the optional addition of 10 × gentamycin at 50 μg/mL (Gibco, New York, United States) (Figure 1E). Tissues were minced meticulously with a scalpel or iris scissors into 1–2 mm^3^ pieces, and digested with 0.1% (w/v) collagenase D solution (Merck KGaA, Darmstadt, Germany) in PBS for 20 h at 37°C in an orbital-motion shaking water bath (Figures 1F–H). After digestion, the collagenase was neutralized with the same volume of growth medium (4,500 mg/L glucose Dulbecco’s modified Eagle’s medium; HG-DMEM (Gibco, New York, United States), supplemented with 10% FBS (Sigma-Aldrich, St Louis, United States) and 1% antibiotic/antimycotic solution), and the tissue pieces were dissociated by pipetting them into a 10 mL pipette. The samples were then centrifuged at 200 × g for 10 min at 25°C to remove the upper fat layer (Figure 1I). The cell suspension fractions were then strained through 100 and 70 μm nylon cell strainers and centrifuged at 3,000 rpm for 5 min at 25°C (without a brake) to collect the cell pellets. Cell pellets were resuspended in the growth medium before plating them into a T75 cm^2^ culture flask (Figures 1J,K). All cultures were maintained at 37°C with 5% CO^2^ in a humidified atmosphere at Passage 0. The growth medium was replaced every 3–4 days. At 80% confluence (around 12–14 days), the medium was removed, and the cells were washed with PBS to remove the residual serum. The cells were detached with 0.125% (v/v) trypsin-EDTA (Biochrom, Berlin Germany), centrifuged, and cryopreserved at a concentration of 1 × 10^6^ cells/ml in a freezing medium containing HG-DMEM, 10% DMSO (Carlroth, Karlsruhe, Germany), and 20% FBS in liquid nitrogen for subsequent analyses.

### Surface Marker Profiling by Flow Cytometry Analysis

The Passage 1 cells were then expanded and cultured by thawing a frozen vial of cells and plating the cells at ∼1.0 × 10^6^ cells/flask or 1.3 × 10^4^ cells/cm^2^ in T75 cm^2^ culture flask. Surface epitopes were then analyzed using Passage 3 cells at ∼80% confluency. Briefly, the cells were washed with PBS and detached with a cell-dissociation buffer (Gibco, New York, United States). After the cells were visibly detached, complete growth medium was added to suspend the cells to analyze the cell viability, which was required to be greater than 90%. Cells were then centrifuged at 3,000 rpm for 10–15 min at 4°C and the supernatant was aspirated. The pellets were washed twice with an ice-cold FACS buffer consisting of PBS with 1% bovine serum albumin (BSA) and 5 mM EDTA (Sigma Aldrich, Louis United States). Subsequently, 100 μl of the cell suspension was pipetted into each Eppendorf tube (5–10 × 10^6^ cells/assay tube) and stained for 60 min at 4°C in the dark with each saturated direct conjugated fluorescein isothiocyanate (FITC)- or phycoerythrin (PE)- primary antibody. The positive conjugated antibodies against CD90, CD105, CD44, integrin beta 1 (CD29), and the FITC- or PE- coupled mouse IgG and IgG2a kappa isotype controls were obtained from Abcam (Cambridge, MA, United States). The negative conjugated antibodies against CD45, CD34, and their mouse IgG1 kappa isotype controls were obtained from eBioscience (San Diego, United States) (Supplementary Table 1). The stained cells were then washed three times with an ice-cold FACS buffer and resuspended with 200 μL of ice-cold FACS buffer and kept on ice until acquisition. Flow-cytometric quantification was performed using an argon-laser-equipped (488 nm) flow cytometer (Gallios, Beckman Coulter). Cells of interest were identified by their size and granularity, as well as the portion of positive cells. The fluorescence intensity (x-mean) of each source was automatically computed. The data analysis was performed using Kaluza software, ver. 1.2 (Beckman Coulter) (Löhrke et al., 2010; Bornhöfft et al., 2020). All analyses were performed on samples from three donors for each synovial tissue source and each breed (*n* = 3 independent biological replicate).

### Differentiation Experiments

Both FP- and FS-derived cells were grown from Passage 1 cryopreserved stocks and the cultures were expanded in T75 cm^2^ culture for third passage until 80% of confluence. The cells were trypsinized, re-seeded at ∼4 × 10^4^ cells/well or 2 × 10^4^ cells/cm^2^ in a 24 well plate. At 70% of confluence cells were subjected to adipogenic induction medium, and at 80–90% of confluence to chondrogenic and osteogenic differentiation medium. The adipogenic induction medium (StemPro^TM^ adipogenesis differentiation kit) and chondrogenic induction medium (StemPro^TM^ chondrogenesis differentiation Kit) were purchased from Thermofisher. The osteogenic differentiation medium contained 10% FBS, 5 μg/mL gentamycin, 100 nM dexamethasone (Sigma-Aldrich, St Louis, United States), 50 μM L-ascorbic acid (Sigma-Aldrich, St. Louis, United States), and 10 mM β-glycerophosphate disodium salt hydrate (Sigma-Aldrich, St. Louis, United States) in HG-DMEM. Adipogenic, osteogenic, and chondrogenic differentiation were induced by culturing both FP- and FS-derived cells from both DL and AS pigs for up to 14, 28, and 35 days, respectively, and the medium was changed twice a week. The differentiation potential of the SMSCs was assessed by histochemical staining and phase-contrast microscopy.

### Histochemistry

After 2–5 weeks of differentiation, the cells in the monolayer were washed with PBS and fixed with 4% formaldehyde in PBS for 30 min. The cells were then rinsed with distilled water, airdried, and stained for histological evaluation. To observe the calcium deposition, osteogenically differentiated cells were stained using 40 mM Alizarin-Red Staining Solution (Sigma-Aldrich, Taufkirchen, Germany) at pH 4.1 for 30 min. Adipogenically differentiated cells were stained using 0.1% Oil Red O solution (Sigma-Aldrich, Taufkirchen, Germany) in 60% isopropanol for 30 min. The chondrogenic potential was evaluated by measuring the production of hyaluronic acid or sulfated mucosubstances produced by chondrogenically differentiated cells. The cells were fixed with an alcohol-based fixative containing with 30% ethanol, 0.4% formaldehyde, and 4% acetic acid in PBS before being stained using Alcian Blue 8GX (Carlroth, Karlsruhe Germany) with 1% Alcian blue solution in a 3% diluted acetic acid solution (pH 2.5) for 30 min. After removing the excess stain, all stained cells were washed thoroughly with distilled water and imaged.

### RNA Isolation and Probe Labeling

For total RNA isolation, the undifferentiated cell pools from all animals (Passage 3, *n* = 6) of each breed (DL and AS) and from each type of synovial tissue (FS and FP) were harvested using a TRI reagent according to the manufacturer’s directions (Sigma-Aldrich, Taufkirchen, Germany), along with an RNeasy kit (Qiagen, Hilden, Germany). They were then treated with DNase and purified using a column-based NucleoSpin RNA II-Kit (Macherey–Nagel, Düren, Germany). RNA integrity was determined by visualization on 1% agarose gel containing ethidium bromide, and the concentration was measured using a NanoDrop ND-1000 spectrometer (PEQLAB, Erlangen, Germany). The absence of DNA contamination was verified by PCR amplification of the porcine *GAPDH* gene (forward primer: 5′-ATGCCTCCTGTACCACCAAC-3′; reverse primer: 5′-AAGCAGGATGATGTTCTGG-3′). All RNA samples were stored at −80°C. To prepare the samples for microarray analysis, 500 ng RNA from each pool was used to amplify the sense-strand cDNA using an Affymetrix GeneChip WT PLUS Reagent Kit (Affymetrix, Santa Clara, CA, United States). The cDNA was fragmented and biotin-labeled using an Affymetrix GeneChip WT Terminal Labeling Kit (Affymetrix, Santa Clara, CA, United States). Each individual sample was hybridized on an affymetrix porcine snowball array (SNOWBALLs520824F) base on Sscrofa11 genome sequence (Affymetrix, Santa Clara, CA, United States) containing 47,880 probe sets. After staining and washing, the arrays were scanned and processed using the Affymetrix GCOS 1.1.1 software.

### Microarray Data Processing, Analyses, and IPA Pathway Analyses

The data were pre-processed using the Affymetrix Expression Console 1.4.1.46 software (Affymetrix), and normalization was performed using the RMA (robust multichip average) expression value (Log2-transformed). A DABG (detection above background) algorithm was used to filter the present (expressed) genes. Probe sets present in less than 75% of the total samples in each breed were excluded from further analysis. Probe sets with a small standard deviation of expression values (≤0.25) across all experimental conditions were filtered out to reduce the number of hypotheses to be tested in the multiple-testing adjustments.

All assessments of porcine synovial membrane tissues were derived from three animals per breed. The means ± SEM were calculated for each measure. Data from the *in vitro* experiments are representative of at least three independent replicate experiments on cells from the same passages. The differential gene expression between breeds, tissues, and their interactions was assessed by running the linear model processes available under Row-by-Row Modeling procedure in the JMP Genomics 9.0 software (SAS Institute, Cary, NC, United States). A *post hoc* Tukey–Kramer test was used for multiple comparison adjustments of all fixed effects. To control for multiple testing, the FDR was set to 0.1. Probe set was defined as a transcript according to the current annotation data of Hadlich et al. (2020). Differentially expressed genes (DEGs) between the different breeds and synovial-membrane-derived MSC sources were submitted to a pathway analysis using the Ingenuity Pathway Analysis (IPA) software (Ingenuity Systems, Redwood City, CA). IPA categorizes genes based on annotated gene functions and statistically tests for the representation of functional terms within the gene list and then calculates adjusted *p*-values using the Benjamini–Hochberg critical value. The microarray data were then deposited in a public database (GEO accession GSE150789).

### Validation of Microarray Results Using Quantitative Real−Time PCR

Same source of total RNA samples used in microarray analysis were used for quantitative real-time PCR (qPCR) to validate the experiments. DNase treated-RNA was reverse-transcribed into cDNA using 200 U of SuperScript II (Invitrogen, Carlsbad, CA) and Oligo (dT) with specific target amplification (STA) and exonuclease I treatment. The master mix for sample inlets consisted of 2.25μL of the STA and Exo-I-treated sample, 2.5μL of SoFast EvaGreen supermix with low ROX (Biorad, Hercules, CA) and 0.25μL of DNA-binding dye. The master mix for assay inlets comprised 2.5μL of assay loading reagent, 2.25μL of DNA suspension buffer, and 0.25μL of a 100μM primer solution (forward and reverse). The qPCR was performed using the BioMark HD Real-time PCR System (Fluidigm, South San Francisco, CA) comprising a 48 × 48 dynamic array with an integrated fluidic circuit for qPCR analyses. All reactions were performed in duplicates along with a no-template negative control (H_2_O control). The qPCR profile conditions consisted of 1 cycle at 95°C with 60 s hold for initial denaturation, followed by 30 cycles of 95°C for 5s and 60°C for 20s. Eight genes (*DKK2, RSPO1, RSPO3, SFRP1, FABP4, NANOG, FGF2*, and *PIK3R1*) were selected, and the primer sequences are available in Supplementary Table 1. *HPRT1, PPIA*, and *YWHAZ* was used as an internal housekeeping control gene and quantifications were carried out using the 2^–Δ^ ^Δ^ ^*Ct*^ methods. Correlation coefficient analysis (r) between the microarray and qPCR data was performed using sas version 9.4 (SAS Institute).

## Results

### Morphology of Fibrous Synovium and Adipose Synovial-Tissue-Derived Mesenchymal Stem Cells

**FIGURE 2:**
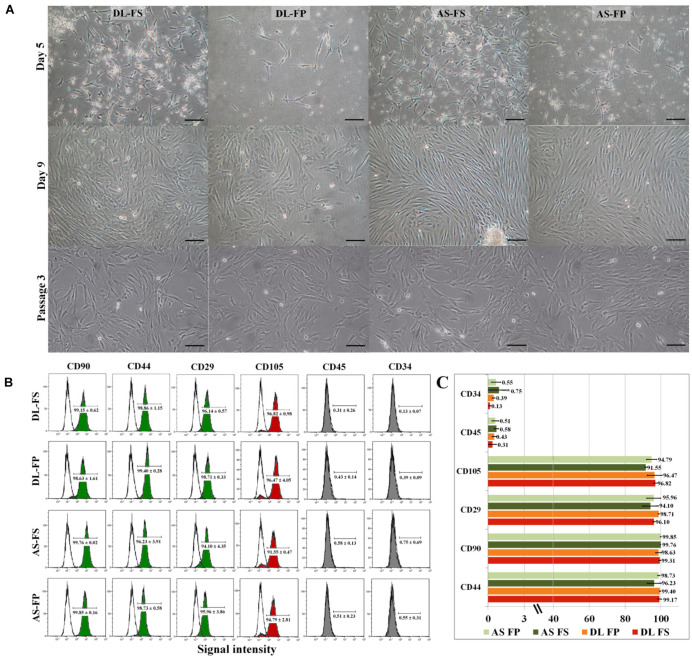
Primary morphology and immunophenotypic profile of the MSCs derived from different synovial tissue sources. **(A)** The adherent cells from FS in both DL and AS breeds started to attach to the plastic surface on Day 2 of the culture and began to proliferate (for FP, this was clearly seen on Day 5 after plating). The cells showed a spontaneous elongated fibroblastic morphology with vortex-like patterns in the confluent area. The cells reached a sub-confluent state in 9–14 days, although other morphologies, e.g., fat-like cells or epithelial-like cells, were observable in all primary cultures. After passaging the cells, both types of cell became relatively homogeneous and showed similar fibroblast-like morphologies. The FS-SMSCs and FP-SMSCs from both DL and AS breeds showed no morphologic differences under a phase-contrast microscope at Passage 3. Scale bars are 100 μm, the same scale for all images. **(B)** Flow-cytometry histograms showing the expression (shaded) of positive cell-surface antigen markers for CD90, CD44, CD29, and CD105 and negative cell-surface antigen markers for CD45 and CD34 molecules for different MSC populations compared with the epitope controls (unshaded peaks), which shared essentially the same surface profile. **(C)** Comparative analysis of the expression of MSC-related markers of DL-FS, DL-FP, AS-FS, and AS-FP derived cells in red, orange, dark green, and light green, respectively. All analyses were performed on cells that were harvested from three animals of each breed. Values are the mean ± SEM percentage expression of each cell-surface protein.

SMSC isolation protocols previously used on dogs and humans were modified for pig tissues. Nucleated cells were obtained from both the FS and FP tissues of the two breeds, DL and AS, and cells were successfully cultured and maintained in a growth medium containing 10% FBS and 1% antibiotic/antimycotic solution. The small spindle-shaped fibroblast-like adherent cells from FS in both the DL and AS breeds began to attach well to the plastic surfaces of the 75 cm^2^ flasks on Day 2 after plating and began to proliferate, while the cells derived from FP were clearly seen nearly 5 days after plating (Figure 2A). The nucleated cells showed a spontaneous elongated fibroblastic morphology and reached a 90% confluent state in 9 days (from FS) and 3–5 days later (Day 12–14) for the FP tissues of both the DL and AS breeds. When they reached 80–90% confluent density, the cells were trypsinized and counted to make a cryopreserved primary cell stock. A comparison between the two types of synovial tissue from both breeds revealed that the primary nucleated cell numbers obtained from the FP tissue were almost 0.5-fold than those of the FS tissue [27 ± 10.4 × 10^6^ cells from DL-FP, 41 ± 12.4 × 10^6^ cells from DL-FS (ratio 1:1.59), 22 ± 4.9 × 10^6^ cells from AS-FP, and 47 ± 3.6 × 10^6^ cells from AS-FS (ratio 1:2.14)]. Other types of morphology, such as fat-like cells or epithelial-like cells, were slightly visible in all primary cultures, but the cells showed similar fibroblast-like morphologies and became relatively homogeneous and uniform after Passage 3, without any morphological differences between the two types of synovial tissue and breeds (Figure 2A).

### Surface Marker Profile of Fibrous Synovium and Adipose Synovial-Tissue-Derived Mesenchymal Stem Cells

Passage 3 of the synovium-derived MSCs was used to verify their purity as stem cells and to characterize their specific surface markers (Supplementary Table 1). The cells derived from the fibrous synovium and adipose synovial tissue of both the DL and AS breeds exhibited similar immunophenotypic characteristics. All cell types had high purity and highly expressed the positive cell-surface proteins of stemness markers, including CD90, CD44, CD29, and CD105 (Figures 2B,C). The cells derived from DL-FS, DL-FP, AS-FS, and AS-FP tissues were observed to be positive (≥90%) for CD44 (98.86 ± 1.15, 99.40 ± 0.28, 96.23 ± 3.91, and 98.73 ± 0.58), CD90 (99.15 ± 0.62, 98.63 ± 1.61, 99.76 ± 0.02, and 99.85 ± 0.16), CD29 (96.14 ± 0.57, 98.71 ± 0.33, 94.10 ± 4.35, and 95.96 ± 3.86), and CD105 (96.82 ± 0.98, 96.47 ± 4.05, 91.55 ± 0.47, and 94.79 ± 2.81), respectively. The cells were also negative (≤1%) and lacked the expression of the negative markers of MSCs: CD45 (0.31 ± 0.26, 0.43 ± 0.14, 0.58 ± 0.13, and 0.51 ± 0.23) and CD34 (0.13 ± 0.07, 0.39 ± 0.09, 0.75 ± 0.69, and 0.55 ± 0.31) (Figure 2C).

### Differentiation Potential of Synovium-Derived MSCs

**FIGURE 3:**
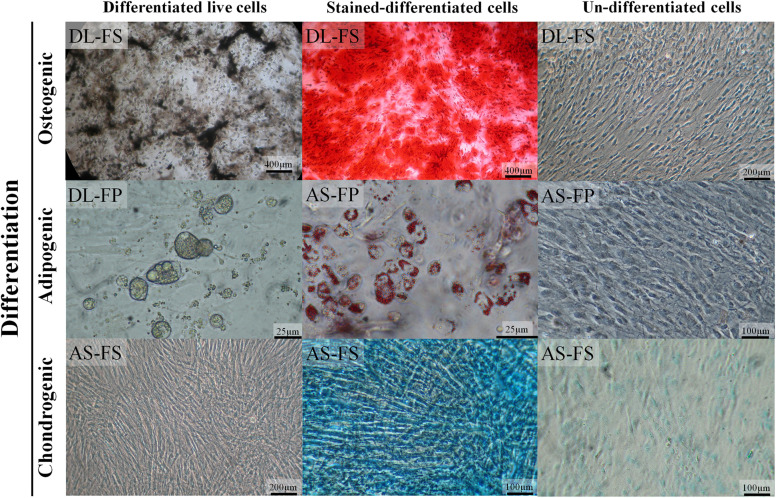
SMSCs derived from different synovial tissue sources in DL and AS breeds successfully differentiated into adipocytes, osteocytes, and chondrocytes under optimal conditions. In the late stages of osteogenesis after culturing in an osteogenic medium for 28 days, calcium deposits were revealed as brown–black lines or spots under a 4× objective phase-contrast microscope and as red-brown after being stained with alizarin red. The lipid-vacuole and lipid-droplet formation of adipocytes was observable at 4 days of adipogenic differentiation; the cells were fixed and stained with Oil Red O to identify the lipid vacuoles (red). For chondrogenic differentiation, the cells were cultivated in a chondrogenic medium for 35 days; sulfated proteoglycans, including hyaluronic acid, were then stained with alcian blue 8GX (bluish-green/blue). The control cultured un-differentiated cells had a fibroblastic morphology under phase contrast similar to that observed before differentiation to chondrocytes, adipocytes, and osteocytes.

The SMSCs derived from both the fibrous synovium and adipose synovial tissue of both the DL and AS breeds successfully differentiated into osteocytes, adipocytes, and chondrocytes (Figure 3). A macrograph of alcian blue 8GX and alizarin red staining as well as bright-field microscopy of lipid-droplet formation were clearly showed the morphological difference between differentiated and non-differentiated (control) SMSCs of each cell type but no differences were noted between different breeds and tissue sources (Supplementary Figure 1). The SMSCs in the osteogenesis stage induced by the osteogenic differentiation medium started to form a non-mineralized collagenous matrix starting at Day 15 of differentiation, and showed calcium mineralization deposits as brown–black lines/spots under a phase-contrast microscope, or as a red-brown color stained with alizarin red from around 21 days, which increased over time. At 14 days after adipogenic induction, the cells showed lipid-droplet deposition under a phase-contrast microscope and positive red lipid-droplet staining with Oil Red–O. The SMSCs also had a tendency to differentiate into elongated polygonal chondrocyte-like cells in the culture system. Bluish-green/blue staining with Alcian blue 8GX revealed sulfated proteoglycans, including hyaluronic acid accumulation, in multilayer cells after chondrogenic differentiation for 5 weeks. Control FS- and FP-derived MSCs cultured with a normal growth medium did not show any evidence of histological differentiation.

### Differentially Expressed Genes

**FIGURE 4:**
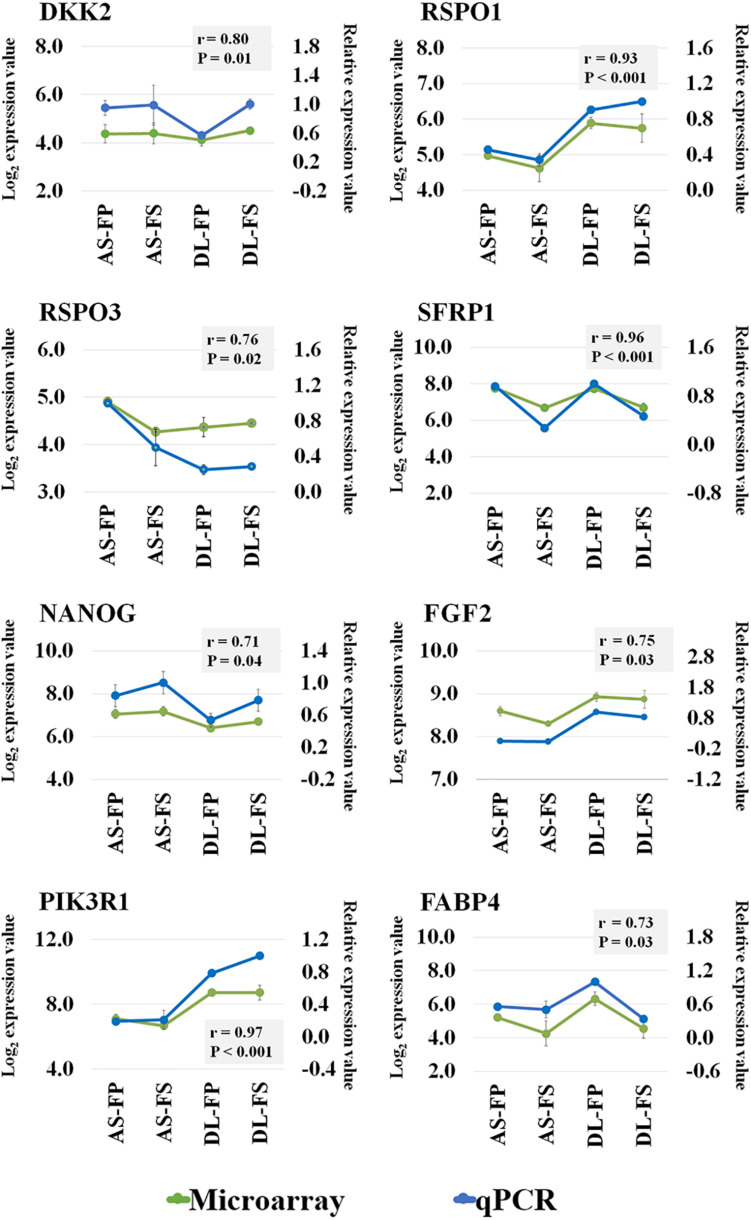
Microarray validation of selected transcripts by RT-qPCR analysis. Correlation coefficient analysis between the microarray and qPCR data for eight genes: *DKK2, RSPO1, RSPO3, SFRP1, NANOG, FGF2, PIK3R1*, and *FABP4* was performed using sas version 9.4. Green line represent the log2 transformed expression value from microarray (the primary *y*-axis) and blue line represent the relative expression value from qPCR (the secondary *y*-axis) for each gene are depicted in the same graph with *r* and *P*-values. *DKK2*; Dickkopf WNT Signaling Pathway Inhibitor 2, *RSPO1*; R-Spondin 1, *RSPO3*, R-spondin 3, *SFRP1*; Secreted frizzled-related protein 1, *NANOG*; Nanog homeobox, *FGF2;* Fibroblast growth factor 2, *PIK3R1;* Phosphoinositide-3-Kinase Regulatory Subunit 1, FABP4; fatty acid binding protein 4.

The Affymetrix Porcine Snowball Array was utilized to assess the transcriptional differences of the SMSCs derived from different breeds and different synovial tissue sources. Expression profiles were compared first between the two breeds, DL and AS, and then between FS and FP samples for each breed. A total of 5,946 out of 47,880 probe sets passed our filtering and were used for the statistical analysis. Selected genes were validated by qPCR as shown in Figure 4. Our transcriptomic data, and qPCR on the same pools of RNA as those used in microarray experiments showed good consistency with the coefficient of correlation (r) ranging from 0.71 to 0.96 among all validated genes.

The number of DEGs (FDR ≤ 0.1), identified through a comparison between different breeds, tissues, and their interactions is shown in Supplementary Table 2. The 191 probe sets (164 genes) were differentially expressed between the AS and DL breeds; a total of 110 probe sets were upregulated in DL, and 81 probe sets were upregulated in AS. In total, 139 probe sets (123 genes) were differentially expressed between the FP and FS tissues; a total of 70 probe sets were upregulated in FP tissue, and 69 probe sets were upregulated in FS tissue. A total of 82 probe sets (75 genes) were differentially expressed between DL-FP and DL-FS, of which 38 probe sets were upregulated in DL-FP, and 44 probe sets were upregulated in DL-FS. Lastly, 86 probe sets (77 genes) were differentially expressed between AS-FP and AS-FS, of which 51 probe sets were upregulated in AS-FP, and 35 probe sets were upregulated in AS-FS.

**FIGURE 5:**
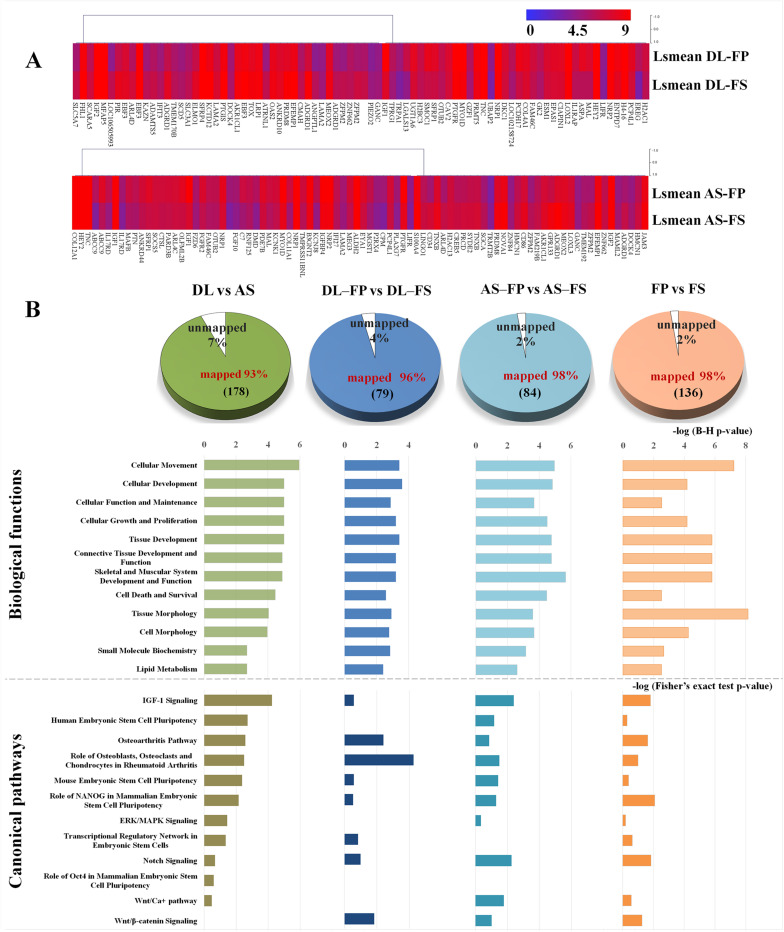
Graphs summarizing results of expression profiling and subsequent functional annotation analyses of SMSC derived from FP and FS of DL and AS breeds. **(A)** Heat-map of differentially expressed genes between FP and FS derived SMSCs within the breeds, DL or AS.; Expression levels are indicated by the color with red color indicating high expression (set to 9) and blue color indicating low expression (set to 0). Genes were clustered according to their transcription patterns using MeV 4.9.0 (Saeed et al., 2003). **(B)** The number of significant DEGs submitted to Ingenuity Pathway Analysis (IPA) and thereof the proportion of un-mapped and mapped transcripts (Supplementary Table 2). Twelve categories of biological functions (light colored bars) and canonical pathways (dark colored bars) enriched for DEGs in the respective comparisons by breed, by synovial tissue type and by or different synovial tissue types in each breed ranked by significance (negative log of B–H multiple testing corrected *p*-values at scale on top of bars or negative log of Fisher’s exact test *p*-values at scale below the bars).

**FIGURE 6:**
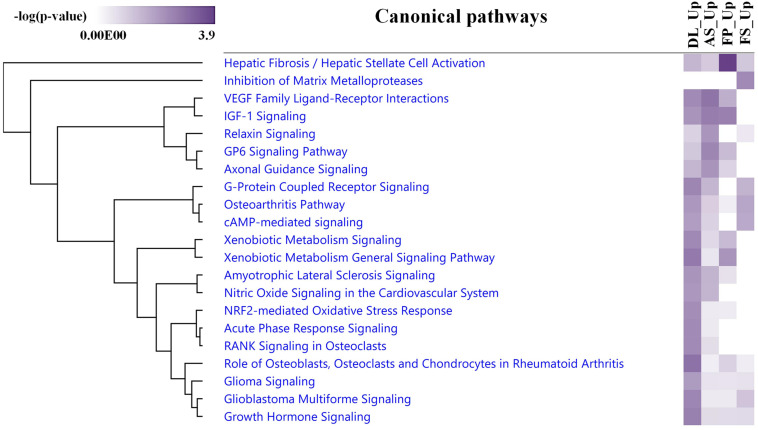
An IPA comparison analysis of the related canonical pathways of DEGs between the upregulated DEGs in each breed and synovial tissue source. Related canonical pathways were hierarchically clustered and displayed with a heat map according to the negative log of the Fisher’s exact test *p*-values.

The average expression levels of differentially expressed genes between DL-FP and DL-FS and AS-FP and AS-FS were visualized using the gene-level heat map (Figure 5A). All significant probe sets were uploaded to IPA to perform a functional analysis. The results of the biofunctional characterization, disease/functional annotation, molecules, and total numbers of genes related to GO-term functions are given in Supplementary Table 3. Focusing on molecular and cellular biological functions related to connective tissues, such as those for skeletal and muscular system development and function, as well as bone, joint, and fat development, were plotted (Figure 5B; lightly painted at the top). The DEGs between AS and DL breeds were significantly enriched in the following five categories: cellular movement, cellular development, cellular function and maintenance, cellular growth and proliferation, and cell death and survival. The SMSCs from DL showed more expressed transcripts enriched in growth hormone signaling and bone metabolism, including the role of osteoblasts, osteoclasts, and chondrocytes, RANK signaling in osteoclasts, and the osteoarthritis pathway (Figure 6).

In contrast, transcripts from the SMSCs of AS breeds were more enriched in VEGF family ligand–receptor Interactions, IGF-1 signaling, relaxin signaling, GP6 signaling, and axonal guidance signaling (Figure 6). Differentially expressed genes between FS and FP tissues were significantly enriched in the following five categories: tissue morphology, cellular movement, connective tissue development and function, skeletal and muscular system development, and function and tissue development (Figure 5B; lightly painted at the top). Notably, the upregulated transcripts from FP tissue showed a high overlap and shared more significant canonical pathways with the SMSCs of AS than DL breeds, including VEGF family ligand–receptor interactions, IGF-1 signaling, GP6 signaling, and axonal guidance signaling (Figure 6). The upregulated transcripts from FS tissue shared more significant canonical pathways with the SMSCs of DL than AS breeds, including G-protein-coupled receptor signaling, the osteoarthritis pathway, and cAMP-mediated dignaling (Figure 6). The DEGs in DL-FP vs. DL-FS and AS-FP vs. AS-FS were significantly enriched in cellular movement, cellular development, cellular growth and proliferation, connective tissue development and function, and tissue development. Of these, 15 genes (*DOCK4, EFEMP1, HEY2, IGF1, IGF2, LAMA2, LIFR, MEOX2, NRP1, NRP2, PRDM8, PTGFR, SFRP1, TNC*, and *ZFPM2*) were the most common DEGs found in these biological functions. Interestingly, *DOCK4, EFEMP1, IGF2, MEOX2, PRDM8*, and *ZFPM2* were highly and specifically expressed in FS tissue, while *HEY2, IGF1, LIFR, NRP1, NRP2, PTGFR, SFRP1*, and *TNC* were found specifically in FP tissue.

All categories of canonical pathway and their associated genes across different comparisons are listed in Supplementary Table 4. Twelve canonical pathways related to stem cell pluripotency, Wnt signaling (and the Wnt pathway), osteoblasts, chondrocytes, ERK/MAPK signaling, and Notch signaling were observed (Figure 5B; below the bar graph in a dark color).

## Discussion

Since the first human synovium-derived mesenchymal stem cells were identified and successfully isolated (De Bari et al., 2001), these cells have been increasingly regarded as a promising cellular source for musculoskeletal regeneration. Besides their general potential to differentiate into various lineages of mesenchymal tissues, SMSCs have a greater ability to expand, proliferate, and superiority in chondrogenesis compared to other MSCs in many species (Fan et al., 2009; To et al., 2019). Several studies have investigated the differentiation potential of MSCs originating from different parts of the same donor to find suitable alternative MSCs sources that best suits their needs and applications (Sakaguchi et al., 2005; Mochizuki et al., 2006; Yoshimura et al., 2007; Alegre-Aguarón et al., 2012; Beane and Darling, 2012; Katagiri et al., 2017; Khatun et al., 2017; Mohamed-Ahmed et al., 2018; Sasaki et al., 2018; Wu et al., 2018). General parameters including surface epitope profiles, proliferative capacity and differentiation potentials of the cells were characterized and compared. Comparative studies indicate the biological differences among MSCs derived from different tissues. Each cell type has both advantages and disadvantages depending upon the research purpose.

Beyond the consideration of the use of procine tissues as excellent large animal model for human in the development of stem cell based therapies, regenerative medicine and transplantation for preclinical research, the generation of porcine MSCs will establish background knowledge and technology in a variety of experimental research for both veterinary clinicians and livestock industry (Swindle et al., 2012; Arrizabalagaa and Nollerta, 2017; Bharti et al., 2016; Markoski, 2016; Schweizer et al., 2020). Application of MSCs may be a powerful tool for treatment of several animal health conditions, and some of the major skeletal abnormalities and disorders that can result in loss of both animal welfare and economic benefits (Fan et al., 2009; Ogata et al., 2015; Gugjoo et al., 2019). Leg weakness and lameness symptoms in pig directly impacts welfare and economic of pig industry, in particular in valuable breeding animals. The relationship between different symptoms of leg weakness and osteochondrosis/osteoarthritis in sows or piglets have been reported (Jørgensen, 2000; Bertholle et al., 2016). Osteochondrosis is a common developmental orthopedic disease affecting both humans and animals. Our previous study showed genome-wide associated studies and functional pathways and networks of candidate genes for osteochondrosis in pigs (Rangkasenee et al., 2013a, b). It might be interesting to perform gain or loss of function *in vitro* experiments of candidate genes by using these porcine SMSCs to differentiate into chondrocytes. Moreover, porcine MSCs are a potential cell source to study bone and cartilage (re-)generation and especially as a model to study functional properties of genes or effects of the vitamin D or phosphorus supplementation on osteogenesis, which is also special area of interest in pigs.

Our study presented a simple and efficient enzymatic digestion method for primary porcine SMSCs isolated from two different sources of synovial membranes of porcine stifle joints for DL and AS breeds, based on to the published synovial mesenchymal stem cell isolation protocols for dogs (Sasaki et al., 2018) and humans (Mochizuki et al., 2006; Katagiri et al., 2017). Comparison among SMSCs from two types of synovial membrane in both breeds demonstrated that they all shared similarities in terms of their cell morphologies, cell-surface marker profiles, and differentiation potential, but differ in their nucleated cell numbers and gene expression profiles. The nucleated cell yield obtained from the FP tissue after expansion was nearly two times lower than the numbers from the FS tissue, which was consistent with the findings of Sasaki et al. (2018) in dogs and confirmed by the suspended synovium culture model in human synovial tissue (Katagiri et al., 2017). In the present study, the nucleated cells isolated from synovial tissue possessed three minimal criteria (Dominici et al., 2006; Bharti et al., 2016): (i) adherence to the plastic surfaces of the culture flasks, (ii) homogeneous appearance and expression of specific surface markers for CD90/CD105/CD44/CD29, but lack of expression of CD45/CD34 and (iii) lastly, a trilineage differentiation capacity following cultivations with certain differentiation media (Dominici et al., 2006; Fellows et al., 2016). All these results confirmed that the nucleated cells were synovium-derived MSCs.

FS and FP synovium-derived MSCs from both DL and AS pigs had similar immunophenotypes, as assessed by cell-surface marker expression. The CD90 and CD44 positive rates from each tissue and breed were over 96%. High expression of CD90 may be related to chondrogenic potential, as previously reported in humans (Nagase et al., 2008). However, the AS-FS-derived SMSCs showed lower expression of the surface marker CD105 at 91.55% ± 0.47% (no statistical significance), possibly due to the fact that a small number of these cells had reduced proliferation, either because the culture started to become confluent or because cells in some areas of the culture did not attach spontaneously at seeding (Anderson et al., 2013; Piñeiro-Ramil et al., 2019). CD105 (enderlin) is a cell-surface glycoprotein identified as a cell proliferation, differentiation, and migration indicator (Fonsatti and Maio, 2004; Anderson et al., 2013). The absence of CD105 expression in mesenchymal stromal cells in mice has been shown to increase osteogenic and adipogenic differentiation (Anderson et al., 2013). However, a lower expression of CD105 does not imply chondrogenic potential in human bone-marrow-derived mesenchymal stem cells (Cleary et al., 2016). It is not clear whether the expression of CD105 or of other specific surface markers, such as CD90, is related to specific lineage differentiation. In our experimental conditions, we did not compare the differentiation potential rate between the SMSCs derived from different synovial tissue sources. This subject would be interesting for further study.

Based on observations made in the present study, SMSCs derived from both the fibrous synovium and adipose synovial tissue of both DL and AS breeds showed similar growth patterns and differentiation potential, and successfully differentiated into osteocytes, adipocytes, and chondrocytes, as shown in previous studies on humans and dogs (Mochizuki et al., 2006; Nagase et al., 2008; Katagiri et al., 2017; Sasaki et al., 2018). Assessment using Oil Red O staining–confirmed that the observed vacuoles were lipids. This method is simple and robust enough to detect adipogenic differences, but its sensitivity must be considered carefully. Lipid vacuoles were observed at ∼4 days of adipogenic induction and increased over time in both size and number until Day 7, with just a slight change afterward. Overall, we found that ∼60–70% of the cells committed to adipocytes, similarly for both types of synovial cell-derived samples from each breed. Our results suggest that prolonging the induction time may not produce a difference in outcome under this differentiation condition. Unlike the results of the adipogenesis assay, optimal osteocyte formation required a minimum of 21 days for all SMSCs, with successful bone mineralization when cultured in DMEM but not αMEM (our preliminary data). The alizarin-red-positive staining of the mineralized calcium matrices produced by SMSCs increased progressively over the culture duration and became abundant in the late stage of osteogenesis in all tissue and breed conditions.

This present study also reported transcriptional differences between the SMSCs from the two breed of pigs. The results showed a number of DEGs that were significantly different by breed, synovial tissue type, and especially between the different synovial tissue types in each breed (FDR ≤ 0.1). A specific stem cell marker, *NANOG*, which participates in all of top five biological function categories (Figure 4), was expressed more strongly in the AS breed than in DL, while no significant difference was found in the expression of other core specific stem-cell transcription factors (i.e., *OCT4* (or *POU5F1*) and *SOX2* (Okumura-Nakanishi et al., 2005) by breed, tissue, or different synovial tissue types in each breed. NANOG is involved in the self-renewal of embryonic stem cells (ES) and is a critical factor for the maintenance of MSC properties (Tsai et al., 2012). Thus, the SMSCs derived from different breeds might have some differences in their cell properties (e.g., proliferation and pluripotency), at least at the transcriptional level. In fact, transcript related to IGF-1 signaling (particularly *PIK3CA*, *PRKAR2B*, and *IGFBP4*) showed higher expression in the SMSCs from AS breeds, while the SMSCs from DL showed greater expression of transcripts, including *IGF2, PIK3R1*, and *SOCS5*, which are enriched in growth hormone signaling. Lists of DEG by synovial tissues types, FP or FS, and of the corresponding enriched pathways revealed that transcripts from FP shared more significant canonical pathways with the SMSCs of AS breeds, while FS tissues shared more significant canonical pathways with the SMSCs of DL breeds. Both findings can be traced back to the original cell donor due to the specific properties of the breeds with DL exhibiting high lean growth and AS being small and more obese (Roberts and Lamberson, 2015). DEGs were also found between the two types of synovial tissues and between breeds (DL-FP vs. DL-FS and AS-FP vs. AS-FS). Notably, DEGs were mainly enriched in cellular movement, cellular development, cellular growth and proliferation, and connective tissue development and function, as well as their tissue development. Moreover, the results of the canonical pathway analysis and the associated genes across different DEG-related comparisons confirmed the differences between breed- and tissue-subtype-derived MSCs in terms of their cell proliferation and specific differentiation efficiency (Rahman et al., 2015; Liskova et al., 2019).

## Conclusion

This study used porcine SMSCs harvested from stifle joints without any complex methods, and may present a routine isolation methodology for many other specific purposes. Higher nucleated cell number obtained from fibrous synovium may be an advantage to harvest the highest amount of SMSCs while, minimizing the amounts of mesenchymal tissues needed. However, these minimal criteria, including cell characteristics and multilineage differentiation potentials, were insufficient to detect the differences between fibrous and obese synovial synovial-derived MSCs that became obvious from the expression analyses. Our study clearly shows the importance of describing the origin of SMSCs in detail, as this has an influence on the results of respective experiments and is necessary to ensure reproducibility. Analyses of the expression and molecular signaling pathways of SMSCs provide additional insights into the functional properties of the cells. Further systematic analyses of differential gene expression as a function of the source of MSCs and direct comparisons during the differentiation processes may lead us to a better understanding of the functional properties and experimental suitability of SMSCs of different niches.

## Data Availability Statement

The microarray data were then deposited in a public database (GEO accession GSE150789).

## Ethics Statement

The animal study was reviewed and approved by the Animal Care Committee of the Leibniz Institute for Farm Animal Biology. Written informed consent was obtained from the owners for the participation of their animals in this study.

## Author Contributions

PS conceived, designed and performed the data analysis, data curation, and writing—original draft. SP: conceptualization, supervision, help analysis data. SP, MO, HR, TV, and KW: writing—review and editing. All authors have read and agreed to the published version of the manuscript.

## Conflict of Interest

The authors declare that the research was conducted in the absence of any commercial or financial relationships that could be construed as a potential conflict of interest.

## Supplementary Material

Supplementary Figure 1 Comparison of the SMSCs differentiation.

Supplementary Table 1 Antibodies used for flow cytometry analysis.

Supplementary Table 2 The number of significant DEGs identified through a comparison between DL and AS breed, between FP and FS tissue, between FP and FS tissue of DL breed and between FP and FS.

Supplementary Table 3 Biological function list of the DEGs between DL and AS Breed, between FP and FS tissue, between FP and FS tissue of DL breed and between FP and FS tissue of AS breed.

Supplementary Table 4 Categories of canonical pathway and their DEGs associated genes between DL and AS Breed, between FP and FS tissue, between FP and FS tissue of DL breed and between FP and FS tissue of AS breed.
