# Supplementary material for: Morphological and Molecular Features of Porcine Mesenchymal Stem Cells Derived From Different Types of Synovial Membrane, and Genetic Background of Cell Donors
Source: Front Cell Dev Biol. 2020 Dec 9;8:601212. doi: 10.3389/fcell.2020.601212 (PMC7755640; doi:10.3389/fcell.2020.601212)
Supplement: Supplementary Figure 1 — Comparison of the SMSCs differentiation. [file Data_Sheet_1.PDF]

Supplementary Material

Supplementary Figures

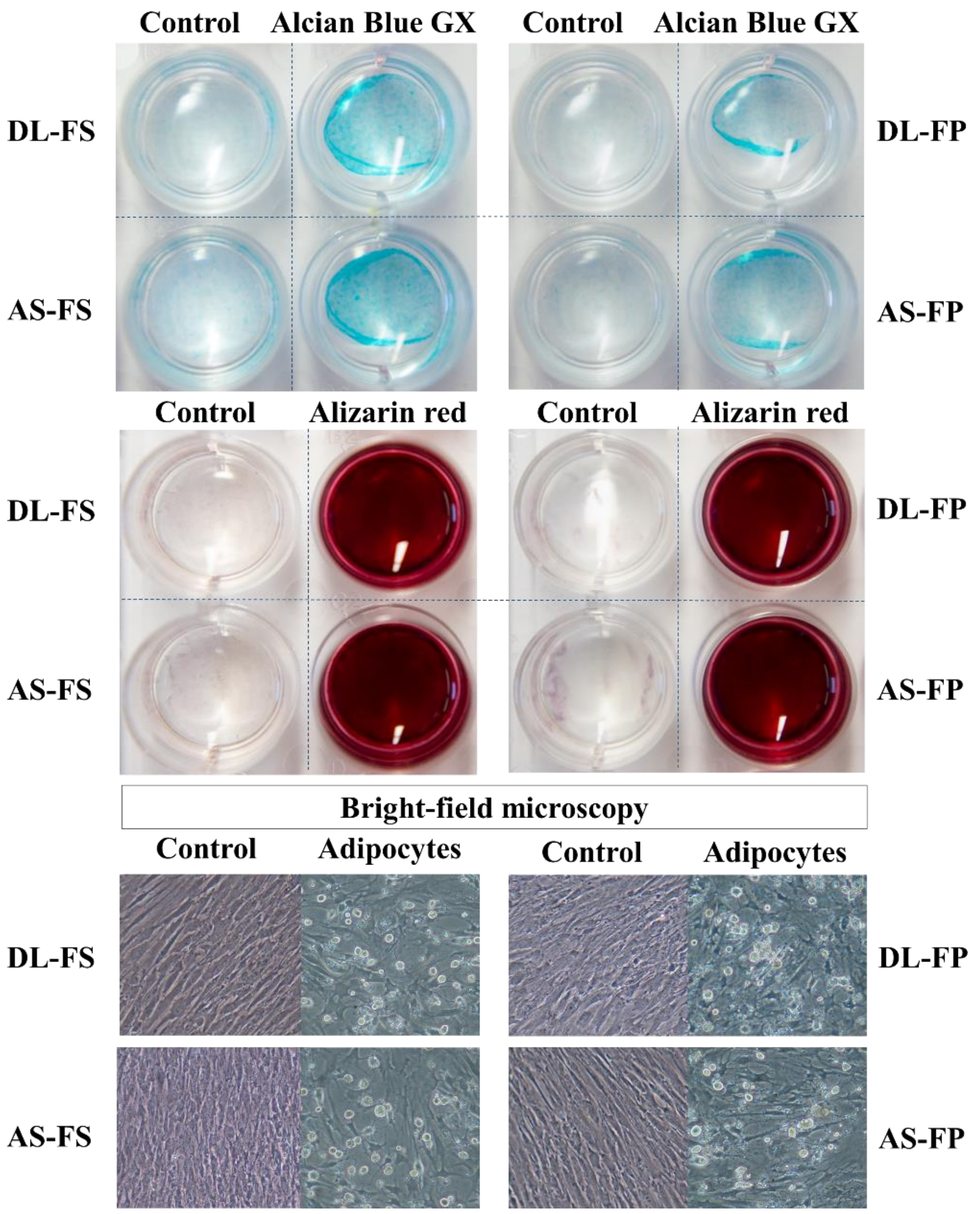

Supplementary Figure S1. Chondrogenic, osteogenic, and adipogenic differentiation of DL-FS, DL-FP, AS-FS, and AS-FP derived SMSCs. The immunohistochemistry macrograph of bluish-green/ blue stained of alcian blue 8GX in the chondrogenic differentiation and red-brown alizarin red stained of osteogenic differentiation as well as bright-field microscopy of lipid-droplet formation in adipocytes clearly showed the difference between differentiated and non-differentiated (control) SMSCs of each cell type.
